# New insights into the pathophysiology and clinical care of rare primary liver cancers

**DOI:** 10.1016/j.jhepr.2020.100174

**Published:** 2020-08-24

**Authors:** Elia Gigante, Valérie Paradis, Maxime Ronot, François Cauchy, Olivier Soubrane, Nathalie Ganne-Carrié, Jean-Charles Nault

**Affiliations:** 1 Service d’hépatologie, Hôpital Avicenne, Hôpitaux Universitaires Paris-Seine-Saint-Denis, Assistance-Publique Hôpitaux de Paris, Bobigny, France; 2 Centre de recherche sur l’inflammation, Inserm, Université de Paris, INSERM UMR 1149 « De l'inflammation au cancer », Paris, France; 3 Service d'anatomie pathologique, Hôpitaux Universitaires Paris-Nord-Val-de-Seine, Assistance-Publique Hôpitaux de Paris, Clichy, France; 4 Service de radiologie, Hôpital Beaujon, Hôpitaux Universitaires Paris-Nord-Val-de-Seine, Assistance-Publique Hôpitaux de Paris, Clichy, France; 5 Service de chirurgie hépato-bilio-pancréatique et transplantation hépatique, Hôpitaux Universitaires Paris-Nord-Val-de-Seine, Assistance-Publique Hôpitaux de Paris, Clichy, France; 6 Université de Paris, Paris, France; 7 Unité de Formation et de Recherche Santé Médecine et Biologie Humaine, Université Paris 13, Paris, France; 8 Centre de Recherche des Cordeliers, Inserm, Sorbonne Université, Université Paris, INSERM UMR 1138, Functional Genomics of Solid Tumors, F-75006, Paris, France

**Keywords:** Mixed tumor, Hepatocholangiocarcinoma, Fibrolamellar carcinoma, Hepatic hemangioendothelioma, Hepatocellular carcinoma, Hepatic angiosarcoma, 5-FU, 5-Fluorouracil, AFP, alpha-fetoprotein, APHE, arterial phase hyperenhancement, CA19-9, carbohydrate antigen 19-9, CCA, cholangiocarcinoma, CEUS, contrast-enhanced ultrasound, cHCC-CCA, combined hepatocholangiocarcinoma, CK, cytokeratin, CLC, cholangiolocellular carcinoma, EpCAM, epithelial cell adhesion molecule, FISH, fluorescence *in situ* hybridisation, FLC, fibrolamellar carcinoma, HAS, hepatic angiosarcoma, HCC, hepatocellular carcinoma, HEH, hepatic epithelioid haemangioendothelioma, HepPar1, hepatocyte specific antigen antibody, iCCA, intrahepatic cholangiocarcinoma, IHC, immunohistochemistry, LI-RADS, liver imaging reporting and data system, LT, liver transplantation, RT-PCR, reverse transcription PCR, SIRT, selective internal radiation therapy, TACE, transarterial chemoembolisation, WHO, World Health Organization

## Abstract

Hepatocholangiocarcinoma, fibrolamellar carcinoma, hepatic haemangioendothelioma and hepatic angiosarcoma represent less than 5% of primary liver cancers. Fibrolamellar carcinoma and hepatic haemangioendothelioma are driven by unique somatic genetic alterations (*DNAJB1-PRKCA* and *CAMTA1-WWTR1* fusions, respectively), while the pathogenesis of hepatocholangiocarcinoma remains more complex, as suggested by its histological diversity. Histology is the gold standard for diagnosis, which remains challenging even in an expert centre because of the low incidences of these liver cancers. Resection, when feasible, is the cornerstone of treatment, together with liver transplantation for hepatic haemangioendothelioma. The role of locoregional therapies and systemic treatments remains poorly studied. In this review, we aim to describe the recent advances in terms of diagnosis and clinical management of these rare primary liver cancers.

**Key points**

• Recent consensus has reclassified pure cholangiolocarcinoma in CCA whereas cHCC-CCA are characterised histologically by the presence of 2 distinct morphological patterns in the same lesion.
• A unique genetic alteration drives the pathogenesis of fibrolamellar carcinoma (*DNAJB1-PRKCA* fusion) and hepatic haemangioendothelioma (*CAMTA1-WWTR1* fusion).
• The combination of imaging and histology, mainly using tumour and non-tumour biopsy, are required for the diagnosis of rare PLCs.
• When feasible, liver resection is the main treatment for rare PLCs.
• No systemic or locoregional therapies are currently validated for the treatment of any unresectable rare PLC.
• Liver transplantation is validated for hepatic epithelioid haemangioendothelioma even in a metastatic setting, whereas this is still an area of research for small cHCC-CCA.

## Background

Hepatocellular carcinoma (HCC) and cholangiocarcinoma (CCA) account, respectively, for 85% and 10% of all primary liver cancers (PLCs). Large cohort studies and randomised controlled trials are available and have enabled the development of international guidelines for the management of HCC and CCA. In contrast, prospective studies and clinical trials are lacking for rare PLCs, such as combined hepatocholangiocarcinoma (cHCC-CCA), fibrolamellar carcinoma (FLC), hepatic epithelioid haemangioendothelioma (HEH) and hepatic angiosarcoma (HAS) due to their scarcity. Herein, we summarise recent advances in our understanding of the pathophysiology of rare PLCs, as well as discussing the latest developments in clinical management.

## Combined hepatocholangiocarcinoma

### A matter of definition

cHCC-CCA is characterised at histology by the presence of 2 distinct morphological patterns in the same lesion: HCC and intrahepatic CCA (iCCA).^1^^,^^2^ Several classifications have been proposed (Table S1)^3^ and were used sequentially in the literature, leading to confusion. The discussion about terminology, based on recent morphological and molecular advances, has led to the exclusion of several types of PLC from the definition of cHCC-CCA: collision tumours, hepatoblastoma, typical HCCs with immunohistochemical expression of progenitor markers, typical iCCAs with immunohistochemical expression of hepatocytic markers. Whether intermediate cell carcinoma and cholangiolocarcinoma (CLC) should be classified as cHCC-CCAs is also debated.

A recent proposal aimed at achieving a consensus terminology^4^ divided PLCs that cannot be defined as either HCC or iCCA into 3 classes:

1. Tumours with hepatocytic and cholangiocytic histology, mixed with a transition or separated areas within the same tumour, which can be considered cHCC-CCA.
2. PLCs completely composed of “intermediate cells” (intermediate cell carcinoma) – small cells of intermediate size (between that of stem cells and hepatocytes) with transitional morphology between hepatocytes and cholangiocytes. Whether these cells can be considered a subtype of cHCC-CCA is still a matter of discussion.^5^^,^^6^
3. PLCs composed of pure CLC; if the main component of PLC is >80% CLC, they can be reclassified as small duct iCCAs.^6^

According to this classification, the concept of “stem cell” phenotypes based on immunohistochemistry (epithelial cell adhesion molecule [EpCAM], cytokeratin (CK)19 and CD56) is not considered as a sub-category *per se*, but rather as a feature that can be present in different types of PLC.^5^ Moreover, all subtypes of PLC could be associated, in the same lesion, with minor histological components observed in more than half of cHCC-CCAs.^7^^,^^8^ If different subtypes are present, a precise description is recommended and the percentage of each tumour type present should be assessed in surgical specimens.^5^

### Epidemiology and risk factors

**Fig. 1:**
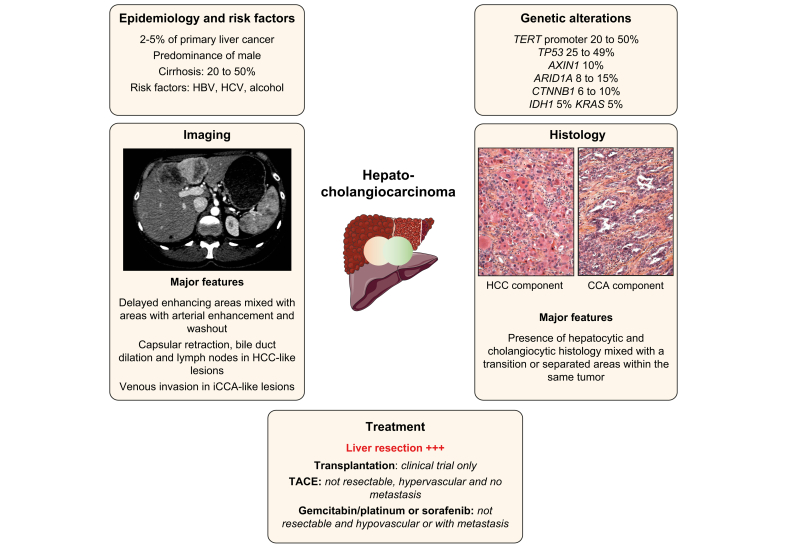
Main characteristics of combined hepatocholangiocarcinoma. Representation of the main genetic alterations, as well as clinical, histopathological, and radiological features of combined hepatocholangiocarcinoma; treatment strategies are also shown. CCA, cholangiocarcinoma; HCC, hepatocellular carcinoma; TACE, transarterial chemoembolisation.

Data from large databases reported that nearly 0.75% of all PLCs are cHCC-CCAs, with an incidence of 0.05/100,000 in the general population.[9], [10], [11] The incidence in monocentric studies based on resection or necropsies varies from 2.4% to 5.3% of PLCs and the 2018 World Health Organization (WHO) classification estimates the frequency at 2%–5% (Fig. 1).^1^^,^^5^^,^^6^^,^[12], [13], [14], [15], [16]

Risk factors associated with cHCC-CCA are shared with other PLCs and include HBV, HCV, alcohol consumption, cirrhosis and male sex (predominance of up to 79%) (Table 1). The association with cirrhosis has been reported in several surgical series in eastern countries (26% to 81%), where it is mostly associated with HBV infection.^5^^,^^13^^,^^15^^,^[17], [18], [19], [20] In Western countries, cHCC-CCA has been associated with cirrhosis in 52% of cases, with HCV the leading cause in Spain (43%) and the USA (23%) and alcohol the leading cause in France (40%).^14^^,^[21], [22], [23] These data are consistent with a systematic review identifying cirrhosis in 51.7% of liver explants or surgical specimens.^19^ It seems that cHCC-CCA stands at the crossroads between iCCA (low rate of cirrhosis and HBV/HCV infection) and HCC (high rate of cirrhosis and HBV/HCV infection) in terms of underlying liver disease.

**Table 1:** Recent data on risk factors of hepatocholangiocarcinoma.

Author	Country	Numbers of patients	Advanced fibrosis	HCV	HBV	Alcohol	Metabolic syndrome
Sasaki *et al.* 2017^27^	Japan	53	14/24 (58%)	9/19 (47%)	9/44 (21%)	2/19 (11%)	3/19 (16%)
Zhou *et al.* 2017^153^	China	144	91/144 (63.2%)	–	101/144 (70%)	29/144 (20%)	–
Xue *et al.* 2019^29^	China	121	54/115 (47%)	2/115 (2%)	89/115 (77%)	–	–
Okumura *et al.* 2020^154^	Japan	89	30/89 (34%)	29/89 (33%)	37/89 (43%)	–	–
Gentile *et al.* 2019^19^	Systematic Review	437	226/437 (52%)	39/437 (9%)	264/437 (60%)	–	–
Wells *et al.* 2015^22^	USA	39	12/39 (31%)	9/39 (23%)	0/39	3/39(8%)	2/39 (5%)
Gigante *et al.* 2019^23^	France	20	10/20 (50%)	1/20 (4%)	3/20 (15%)	8/20 (40%)	6/20 (30%)
De Martin 2020∗^55^	France	31	31/31 (100%)∗	–	–	40/75 (53%)	–
Holzner 2020^54^	USA	47	20/47 (43%)	15/47 (32%)	22/47 (47%)	–	–

We included recent studies with histologically confirmed (Goodman transitional type (type II)/Allen and Lisa type B or C/WHO classical type tumours and stem cell type with exception of CLC, studies already included in the systematic review (Gentile *et al.* 2019) are not shown.

∗ Study including only lesions on cirrhosis. Data about risk factor prevalence are relatives to the entire cohort of cHCC-CCA and iCCA.

### Genetic landscape of cHCC-CCA

A whole-genome sequencing analysis of liver cancers displaying biliary phenotype, including cHCC-CCA, reported a median number of 60 to 70 non-synonymous coding mutations per tumour (Table 2).^24^ *TERT* promoter, *TP53*, *ARID1A* and *ARID2* mutations are more frequent in cHCC-CCA; *PBRM1*, *BAP1*, *KRAS*, *IDH1* and *FGFR2* mutations in iCCA; and *CTNNB1* mutations in HCC (Fig. 1). The high heterogeneity in terms of techniques and HCC-CCA classification in these studies needs to be underlined.[25], [26], [27], [28] Genomic analysis also suggests an impact of viral hepatitis (HCV and HBV) on the genetic landscape of cHCC-CCA that seems closer to HCC than iCCA in terms of genomic profiles and prevalence of *TERT* promoter mutations.^24^

**Table 2:** Genomic alterations in rare primary liver cancers.

Study	Classification	Type of analysis	N patients Subtypes	Fibrosis (F3-F4)	Somatic genetic alterations
**Hepatocholangiocarcinoma**					
Cazals-Hatem *et al.* 2004^155^	Lisa et Allen 1949	Target sanger sequencing	14 mixed, 1 fibrolamellar HCC 3 collision tumours	3/15	*TP53*
Fujimoto *et al.* 2015^24^	WHO 2010	WGS and RNA-seq	30 Liver cancer with biliary phenotype 7cHCC-CCA +2CLC	4/9	*TERT* promoter 53%, *PBMR1* 20%, *ARID2* 27%
Sasaki *et al.* 2017^27^	WHO 2010	Target sanger sequencing + IHC	53 mixed tumours 4 CT, 4 TS, 20 INT, 25 CLC	38/53	cHCC-CCA: *TERT* 50%, *TP53* 25%, *KRAS* 50% *ARID1A* 0% Intermediate: *TERT* 42%, *TP53* 58%, , *KRAS* 5%, *ARID1A* 11%
Moeini *et al.* 2017^28^	WHO 2010	Microarray, DNA copy number, WES	18 mixed tumours 6 CLC/8SC/4CT	10/18	CLC: *TP53* and IDH1 cHCC-CCA: *TP53*, *TERT* promoter, *BRAF*, *FGFR2-BICC1* fusion
Liu *et al.* 2018^25^	WHO 2010	WGS, WES and RNA-seq	4 cHCC-CCA not specified	n.a.	*TP53*, *CTNNB1* and *ARID1A*
Wang *et al.* 2018^31^	WHO 2010	WES	7 cHCC-CCA	n.a.	*TP53* and *ARID2*
Xue *et al.* 2019^29^	Lisa et Allen 1949	WES, WGS, RNA-seq,	121 tumours: 6 separate type, 56 combined type, 59 mixed type.	54/115	*TP53* 49% , *TERT* promoter 23%, *AXIN* 10%, *KMT2D* 9%, *KEAP1* 8%, *ARID1A* 8%, *RB1* 8%, *CTNNB1* 6%, *IDH1* 5%
Joseph *et al.* 2019^26^	Consensus 2019	Target next-generation sequencing	20CT	15/18	*TP53 (80%), TERT (70%), ARID1A (15%), CTNNB1 (10%), AXIN1 (10%), KRAS (5%)*
Sasaki *et al.* 2019^30^	Consensus 2019	Target sequencing + IHC	9 CT	6/9	*TP53* (66%), *TERT* promoter (33%), *KRAS* (22%)
**Fibrolamellar carcinoma**					
Honeyman *et al.* 2014^79^	n.a.	RNA-seq	15 FLC	0	*DNAJB1-PRKACA* fusion (100%)
Cornella *et al.* 2015^80^	n.a.	FISH WES	78 FLC	0	*DNAJB1-PRKACA* fusion (79%) *BRCA2* (4.2%)
Graham *et al.* 2015^81^	n.a.	RT-PCR FISH	26 FLC	0	*DNAJB1-PRKACA* fusion (100%)
Graham *et al.* 2018^86^	n.a.	FISH NGS	3 FLC without *DNAJB1-PRKACA* fusion	0	*PRKAR1A (100%)* in patients with Carney syndrome and FLC
Graham *et al.* 2018^156^	n.a.	FISH	104 typical FLC, 12 probable FLC and 9 unlikely FLC	0	*99% DNAJB1-PRKACA* fusion in typical, 75% in probable and 0% in unlikely FLC
**Hepatic haemangioendothelioma**					
Tanas *et al.* 2011^113^	n.a.	RNA-seq FISH	47 haemangioendothelioma (hepatic and non-hepatic)	0	89% *WWTR1-CAMTA1* fusion
Errani *et al.* 2011^115^	n.a.	FISH	17 haemangioendothelioma (hepatic and non-hepatic)	0	100% *WWTR1-CAMTA1* fusion
Antonescu *et al.* 2013^116^	n.a.	FISH	10 haemangioendothelioma without *WWTR1-CAMTA1*	0	100% *YAP1-TFE3* fusion (in tumours without WWTR1-CAMTA1 fusion)
Flucke *et al.* 2014^157^	n.a.	FISH RT-PCR	35 haemangioendothelioma (hepatic and non-hepatic)	0	94% *WWTR1-CAMTA1* and 6% *YAP1-TFE3* fusion
Patel *et al.* 2015^117^	n.a.	RT-PCR	18 haemangioendothelioma (hepatic and non-hepatic)	0	78% *WWTR1-CAMTA1* and 6% *YAP1-TFE3* fusion

Molecular alterations of HAS were not represented as very few data are currently available in the literature. CLC, cholangiolocarcinoma; CT, classical type; IHC, immunohistochemistry; INT, intermediate subtype; SC, stem cell subtype; TS, typical subtype; WES, whole-exome sequencing; WGS, whole-genome sequencing.

The largest genetic study on cHCC-CCA was performed on 133 patients in Asia. Similar to the genomic alterations observed in HCC, *TP53*, *TERT* promoter, *AXIN1*, *KMT2D*, *ARID1A* were the most common mutations in cHCC-CCAs; the frequency of *TERT* promoter mutations (23%) was lower than in HCCs (40–60%) but higher than in iCCAs (0–8%). The analysis of mutational signatures identified an exposure to aristolochic acid, aflatoxin B1 and hepatitis B. Epithelial-mesenchymal transition, EpCAM, and KRT19 genes were mostly expressed in “combined” type cHCC-CCA with an enrichment of *KRAS* mutations. Xenobiotic and bile acid metabolism and overexpression of alpha-fetoprotein (AFP), glypican 3, and spalt-like transcription factor 4 were more frequently observed in “mixed” type cHCC-CC according to the Allen Classification. The authors suggest that “mixed” type cHCC-ICC could be more similar to HCC and “combined” type cHCC-CCA more similar to iCCA.^29^

Analysis of genetic landscapes shows that CLC has a different genetic profile to pure cHCC-CCA with more *ARID1A* and less *TERT* promoter mutations.^30^ Another genomic analysis confirms that CLC looks like a biliary-derived molecular entity harbouring chromosomal stability and activation of TGFβ pathway with biliary features.^28^

In terms of tumour heterogeneity, comparing the iCCA and HCC components confirms the monoclonal origin of cHCC-CCA but also shows a significant intratumor genetic heterogeneity that overlaps with morphological heterogeneity.^29^^,^^31^ One study identified *TERT* promoter mutations in both HCC and iCCA components suggestive of an early event in carcinogenesis, whereas mutations in other driver genes such as *TP53* harboured intratumoural heterogeneity.^26^

In terms of the cell of origin, the disruption of p53 in mice promotes dedifferentiation of mature hepatocytes into nestin-positive progenitor cells that could give rise to HCC or iCCA under the influence of Wnt and Notch.^32^^,^^33^ Overexpression of nestin was identified in 81.3% of human cHCC-CCAs and was associated with a poor clinical outcome.^29^ Moreover, a cell line derived from cHCC-CCA can differentiate into either HCC or iCCA under different growth conditions.^34^^,^^35^ These results are consistent with the hypothesis that cHCC-CCA can derive from hepatic progenitor cells that express markers of both lineages (hepatocytes and biliary cells).^36^^,^^37^

These data suggest that i) cHCC-CCA is monoclonal, deriving from a common cell of origin; ii) cHCC-CCA genomic features may be more similar to HCC than iCCA, even if some cHCC-CCA harboured genomic features closer to iCCA; iii) risk factors can be associated with specific genetic features in cHCC-CCA; and iv) CLC has a different molecular profile that is similar to iCCA.

### Diagnosis

**Fig. 2:**
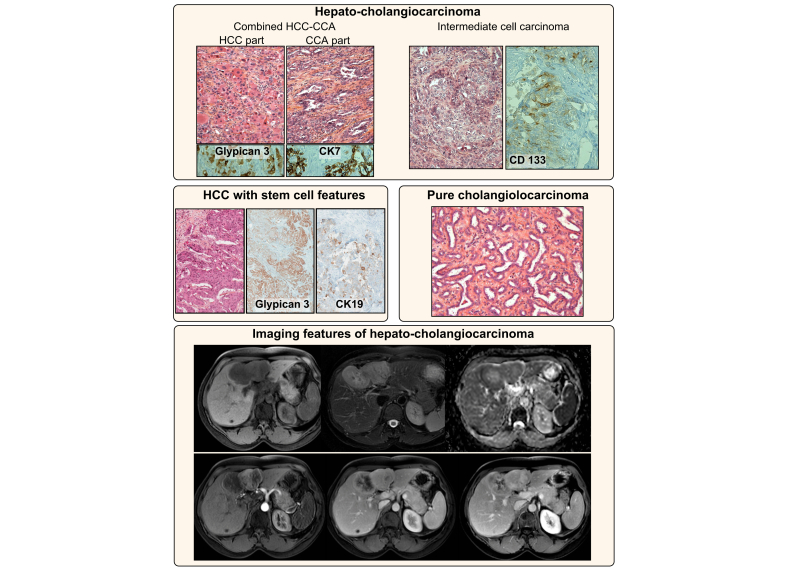
Histological and radiological features of hepatocholangiocarcinoma. Classical cHCC-CCA (upper panel, left) with the HCC (positive for glypican 3) and CCA (positive for CK7) components, as well as intermediate cell carcinoma (positive for CD133) (upper panel, right). HCC with stem cell features (middle panel, left) at immunohistochemistry (positive for glypican 3 and for CK19) and cholangiolocarcinoma recently reclassified as iCCA (middle panel right). An example of an MRI of cHCC-CCA with a well-delineated heterogeneous lesion with capsular retraction. The lesion harboured progressive delayed enhancing areas mixed with areas with arterial enhancement and washout. cHCC-CCA, combined hepatocholangiocarcinoma; CCA, cholangiocarcinoma; HCC, hepatocellular carcinoma.

The diagnosis of cHCC-CCA is based on histology from biopsies or surgical specimens (Fig. 2).^5^^,^^6^ Immunohistochemical markers are not mandatory but can be helpful to better characterise PLCs: hepatocyte markers (HepPar1, AFP and glypican 3); cholangiocyte markers (CK19, CK7) and “stem cell” markers (EpCAM, CK19, CD133).^5^^,^^23^ These markers should be considered in the context of morphological analysis, especially “stem cells markers” that could be expressed by all PLCs. In the pre-surgical setting, liver biopsy had an estimated 48% sensitivity and 100% specificity for the diagnosis of cHCC-CCA.^23^

Sometimes the discordance between imaging and serum tumour markers (imaging suggestive of HCC with increased serum carbohydrate antigen 19-9 [CA19-9] or hypovascular nodule suggestive of iCCA with increased AFP) could raise the suspicion of a cHCC-CCA.^13^^,^^18^ However, serum biomarkers alone are not reliable for the diagnosis of cHCC-CCA, with elevation of serum CA19-9 and AFP only observed in 45% of cases and with limited specificity.^22^^,^^38^

Even though histology remains the gold standard for the diagnosis of cHCC-CCA, radiology (abdominal CT or MRI with contrast agent injection) may help guide the diagnosis (Fig. 2). Hallmarks of HCC (arterial phase hyperenhancement [APHE] and washout) are observed in a minority of cHCC-CCAs.^22^^,^^23^^,^^39^ Nevertheless, recent studies using the American College of Radiology's liver imaging reporting and data system (LI-RADS) have reported misclassification of cHCC-CCA as HCC in 26% to 54% of cases when using major radiological features.^40^^,^^41^ Notably, 88% of these patients could be reclassified as having malignant tumours that are not HCC (LI-RADS M category) after addition of ancillary features such as rim/peripheral APHE, progressive central enhancement on portal venous and delayed phase images, predominantly peripheral washout appearance, liver surface retraction, biliary obstruction and marked diffusion restriction.^40^ The depiction of these features explains why the main differential diagnosis is often iCCA, and why performance of imaging is often insufficient.^39^^,^[42], [43], [44], [45], [46] The association of HCC features with CCA features (appearance of iCCA with portal venous invasion, or appearance of HCC with biliary dilation or enlarged lymph nodes) may guide the diagnosis. Finally, contrast-enhanced ultrasound (CEUS) also harbours an insufficient specificity for the diagnosis of cHCC-CCA, since tumours exhibit various degrees of heterogeneous APHE with washout.^47^^,^^48^

Imaging has a limited diagnostic performance alone, with a sensitivity of only 48% and a specificity of 81%, though the combination of imaging and biopsy can improve the sensitivity (60%) and specificity (82%).^23^ Overall, radiology is fundamental to guide liver biopsy (especially possible multiple biopsies in heterogeneous tumours) and to perform tumour staging.

### Treatments

#### Liver resection

Liver resection is currently the most effective curative-intent therapy for cHCC-CCA. According to state-of-the-art principles for oncologic liver surgery, liver resection aims to completely remove the lesion with adequate margins and with a sufficient liver remnant volume. This requires a multi-parametric evaluation of the patient, tumour and underlying liver disease.^49^ A resection margin >10 mm has been associated with prolonged disease-free survival.^50^ Major hepatectomy can be proposed if a sufficient liver remnant volume has been secured in order to limit the risk of postoperative liver failure.^49^ In patients with cirrhosis, evaluation of the degree of portal hypertension should also be performed as clinically significant portal hypertension represents an absolute contraindication to major hepatectomy.^51^ Furthermore, a lymphatic pattern of tumour spread in cHCC-CCA requires a routine hilar lymphadenectomy.^52^ The need for routine lymphadenectomy should currently restrict the use of the laparoscopic approach only to centres with extensive expertise both in liver surgery and laparoscopy.^53^

In a systematic review that included 437 patients with cHCC-CCA, liver resection led to an average disease-free survival of 14.2 months in patients with cHCC-CCA, 43.1 months in those with HCC, and 17.8 months in those with iCCA, corresponding to an average overall survival of 37, 67 and 32 months, respectively.^19^ Outcomes after liver resection for cHCC-CCA are similar to those for iCCA and worse than in patients with HCC, mainly due to early tumour recurrence,^14^^,^^18^ although a recent study identified no difference in outcomes after adjustment for cirrhosis and tumour size.^54^

#### Liver transplantation

The role of liver transplantation (LT) in the treatment of small iCCA or cHCC-CCA remains controversial. A systematic review of retrospective studies on LT for cHCC-CCA reported a median disease-free survival of 14.2 months and a median overall survival of 37.1 months.^19^ These results were discouraging and, in many countries, cHCC-CCA is still a contraindication for LT.

In contrast, recent studies with similar inclusion criteria reported more positive outcomes. The first study was conducted in Spain on 42 patients undergoing LT for HCC, with an incidental diagnosis of HCC-CCA or iCCA, who were stratified according to tumour size and number. The 5-year survival rates were similar between cHCC-CCA and HCC controls (78% *vs.* 86%). Patients with multinodular or uninodular tumours larger than 2 cm had the worst outcomes.^21^ The second retrospective study analysed patients treated by resection (n = 26) or LT (n = 95) for iCCA and cHCC-CCA <5 cm developed on cirrhosis. Overall survival (67% at 5 years) and recurrence-free survival (75% at 5 years) were better in patients treated by LT than in patients treated by resection. Survival was similar in patients with iCCA or cHCC-CCA.^55^

Recent retrospective data suggest that transplantation improves survival compared to resection in cirrhotic patients with cHCC-CCA, if tumour size is <5 cm.^21^^,^^55^^,^^56^ One of the main drawbacks of these studies is that cHCC-CCA was identified incidentally on the explant and intention-to-treat analyses of LT for cHCC-CCA diagnosed before inclusion on the waiting list are lacking. A recent consensus concluded that there is not enough evidence to propose LT for cHCC-CCA but that this approach should be explored in clinical trials.^57^ Moreover, LT should also be discussed according to national guidelines.

#### Locoregional treatments

The effectiveness of transarterial chemoembolisation (TACE) on cHCC-CCA has been analysed in retrospective studies in a limited number of patients. In a study of 50 patients, TACE induced a partial response or stable disease in 70% of cases, mainly in tumours with APHE, leading to a median overall survival of 12.3 months.^58^

Better outcomes were reported in a cohort of patients treated by TACE for recurrence after liver resection. As expected, cHCC-CCAs with a non-rim APHE pattern at imaging are associated with a better radiological response rate (36% *vs.* 0%) and survival (52.8 *vs.* 12.4 months) compared to tumours with a rim APHE pattern.^59^^,^^60^ Data on radio-embolisation (selective internal radiation therapy [SIRT]) and chemotherapy for unresectable iCCA show that 22% of patients can be downstaged for surgical intervention.^61^ In 1 study, SIRT was associated with a 55% radiological response rate, 65% disease control rate, and a median overall survival of 9.3 months in 21 patients, suggesting a possible role for SIRT in cHCC-CCA.^62^

Altogether, few data are currently available to support the value of intra-arterial treatments in patients with cHCC-CCA, even if some retrospective data suggest a possible role in selected patients with tumours showing APHE.

#### Systemic treatments

Data on systemic treatments for unresectable cHCC-CCA are limited to retrospective series testing the first-line treatments approved for advanced HCC (sorafenib) and CCA (gemcitabine/platinum regimens).^63^^,^^64^

A multicentre Japanese study in 36 patients with unresectable cHCC-CCA analysed different first-line systemic treatments. The median overall survival with gemcitabine/cisplatin, fluorouracil/cisplatin and sorafenib was 11.9, 10.2 and 3.5 months, respectively, suggesting that sorafenib was associated with reduced survival.^65^ A French multicentric study included 30 patients treated with gemcitabine and oxaliplatin or cisplatin ± bevacizumab. Eight patients (28.6%) had a partial response with a median progression-free survival of 9.0 months and an overall survival of 16.2 months.^66^ The largest series available was a monocentric cohort of 68 patients with unresectable cHCC-CCA who received mainly gemcitabine-based regimens (57/68), of whom 23.5% received gemcitabine ± fluoropyrimidine and 60.3% gemcitabine with platinum. Overall survival was 11.5 months in patients receiving gemcitabine/platinum therapy and 9.6 months in the 7 patients treated with sorafenib alone.^67^ Currently, no data are available regarding the use of atezolizumab/bevacizumab, lenvatinib, cabozantinib and ramucirumab in cHCC-CCA.

To summarise, systemic treatments based on gemcitabine/platinum regimens are the most widely used, but their use is not supported by a high level of evidence. The role of sorafenib remains unknown.

### Fibrolamellar carcinoma

**Fig. 3:**
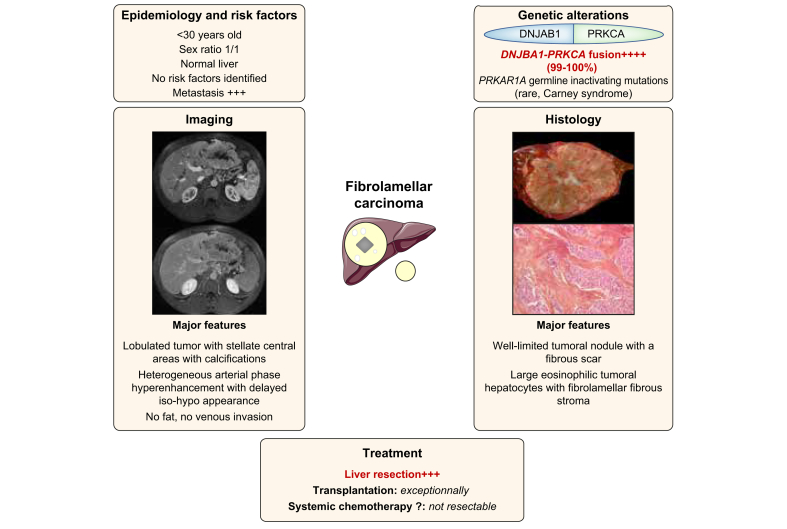
Main characteristics of fibrolamellar carcinoma. Representation of the main genetic alterations, as well as clinical, histopathological, and radiological features of fibrolamellar carcinoma; treatment strategies are also shown.

FLC is a rare PLC derived from hepatocytes that occurs in young adults (sex ratio 1:1) on normal liver (Fig. 3).[68], [69], [70], [71], [72], [73] It is characterised by eosinophilic polygonal cells and prominent nucleoli, with fibrotic tissue surrounding tumour cells on histology.^68^^,^^73^^,^^74^ No risk factors for FLC development have been identified so far. Most FLCs are diagnosed before 40 years, with the median age of diagnosis ranging from 20–29 years.[77], [78], [79] FLCs are larger (9-13 cm) with a higher rate of lymph node invasion (43–46%) compared to HCC.^71^^,^[75], [76], [77] The most frequent sites of metastases are the lung (50%), bone (19.2%), and brain (1.9%).^78^

#### Pathophysiology

At the molecular level, *DNAJB1-PRKCA* fusion – due to a focal deletion in the chromosome 19 – is identified in almost all FLCs and is considered highly specific but not pathognomonic (Table 2).[79], [80], [81] The same fusion was identified in intraductal oncocytic papillary neoplasms of the pancreas and bile duct.^82^^,^^83^ A subset of HCC with fibrolamellar-like features has been shown to occur in non-cirrhotic livers, but in older patients, and was characterised by both *BAP1* alterations and an aberrant activation of the protein kinase A pathway due to a chromosome gain of *PRKACA* combined with a loss of PRKAR2A (the inhibitory regulatory subunit of protein kinase A).^84^ These tumours also expressed neuroendocrine and pancreatic markers pointing to a potential hepato-pancreatic progenitor. Finally, *GNAS* mutations leading to protein kinase A activation were observed in a subset of hepatocellular adenomas with a fibrous stroma.^85^ All these data suggested that protein kinase A activation in the liver was associated with “fibrolamellar-like” features and underlined a link between the activation of protein kinase A and a hepato-pancreatic progenitor lineage. Finally, rare cases of FLC arising in patients with Carney complex were related to germline inactivating mutations in *PRKAR1A* (Table 2).^86^ PRKCA from the *DNAJB1-PRKCA* fusion has a conserved tyrosine kinase domain and an enhanced cAMP-stimulated protein kinase A activity. It leads to a constitutive activation of the protein kinase A pathway and promoted the malignant transformation of hepatocytes in a mouse model.^87^^,^^88^ As *DNAJB1-PRKACA* fusion is a genetic footprint of FLC, it could be used to confirm the diagnosis of FLC using fluorescence *in situ* hybridisation (FISH) or reverse transcription PCR (RT-PCR) in clinical practice.^81^^,^^89^

#### Diagnosis

Most of the time diagnosis is made in a symptomatic patient with abdominal pain and weight loss.^74^ Rarely, obstructive jaundice, gynecomastia in males, encephalopathy, ascites, acute liver failure, recurrent thrombophlebitis, anaemia, hypoglycaemia or Budd-Chiari syndrome can reveal FLC.[90], [91], [92] Differential diagnosis consists of primary liver tumours with fibrosis, such as some subtypes of HCC (especially *BAP1* mutated HCC), CCA or focal nodular hyperplasia.

The diagnosis of FLC could be suspected on CT and MRI based on the clinical context (young patient without chronic liver disease). FLCs are usually large and lobulated heterogeneous lesions with a central stellate scar seen in 65–70% of cases and tumour calcifications and abdominal lymphadenopathy observed in half of cases.^93^^,^^94^ On MRI, FLC show T1-weighted hypointensity and T2-weighted hyperintensity with a central area showing hypointensity on both T1- and T2-weighted images.^93^ FLCs exhibit heterogeneous APHE, with a variable enhancement pattern on portal venous and delayed phases.^91^ Noticeably, FLCs never contain fat, and do not invade hepatic or portal veins in contrast to classical HCC. FLCs are also hypointense on the hepatobiliary phase (using hepatobiliary contrast agents).^95^ FLCs do not usually produce detectable AFP and less than 10% of patients have AFP levels above 200 ng/ml.^90^

Tumour and non-tumour liver biopsy is usually advised in clinical practice, with the exception of patients eligible for front-line surgery irrespective of the results of biopsy.^92^^,^^96^ High rates of CK7- and CD68-positive staining on liver samples and low rates of glypican 3-positive staining could differentiate FLC from regular HCC.^97^^,^^98^

#### Treatment

In a systematic review including 575 patients, those treated with partial hepatectomy (55%) had 5-year overall survival rates of 70%.^77^ Liver resection was associated with a better overall survival in patients with FLC compared to patients with classical HCC (median overall survival of 84.9 *vs.* 42.9 months, respectively). However, no significant difference in 5-year survival could be observed when focusing on patients without cirrhosis, suggesting that the difference observed in the overall population was likely related to the severity of the underlying liver disease.^70^^,^^72^^,^^78^ Currently, liver resection remains the most effective curative-intent treatment option for FLC; aggressive initial surgical resection along with regional lymphadenectomy is advised.^76^^,^^77^

In contrast, results of LT are impaired by a high rate of tumour recurrence leading to a 5-year overall survival of 35%.^77^ However, the absence of selection criteria for patients treated by LT limits the interpretation of these studies.^72^^,^^77^^,^^99^ Slightly better results were recently reported in 63 patients undergoing LT, with an overall survival rate of 48% at 5 years.^100^ As for other indications, LT should be discussed according to national guidelines.

Patients with unresectable disease (20 to 25% at diagnosis) are treated with various combinations of systemic therapy, with or without locoregional therapies. The role of TACE or SIRT alone is also poorly studied. Chemotherapy regimens included 5-fluorouracil (5-FU) + cisplatin or irinotecan, doxorubicin and gemcitabine + oxaliplatin, but few patients exhibited a radiological response.^101^ Sorafenib was associated with stable disease in 4 out of 9 patients and 1 patient achieved a complete response with an anti-PD1 antibody.^102^ Moreover, aurora kinase A inhibitors had a limited antitumour effect in a phase II clinical trial.^103^ Shutdown of the PRKCA pathway and targeting the *DNABJ1-PRKACA* fusion is an appealing therapeutic avenue. While several therapeutic options have been proposed in FLC, such as inhibitors of the kinase pocket of the fusion protein or the combination of Hsp70 and MEK inhibitor,^104^^,^^105^ currently no efficient targeted therapy has been validated.

### Hepatic epithelioid haemangioendothelioma

**Fig. 4:**
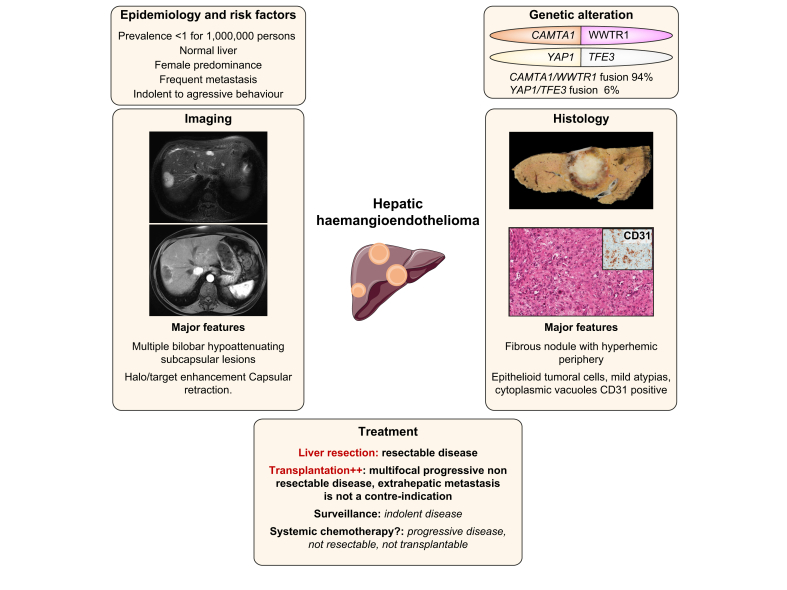
Main characteristics of hepatic haemangioendothelioma. Representation of the main genetic alterations, as well as clinical, histopathological, and radiological features of hepatic haemangioendothelioma; treatment strategies are also shown.

HEH is a rare vascular tumour that develops on normal liver, characterised by epithelioid and histiocytoid vascular endothelial cells in a fibrotic stroma (Fig. 4).^106^ Tumour cells are positive for endothelial markers (factor VIII-related antigen, CD34 and CD31) on immunohistochemistry.^106^ Tumour cells likely invade pre-existing vascular channels including centrilobular veins at the periphery. Some risk factors have been suggested in the literature such as oral contraception, vinyl chloride, thorotrast, asbestos, or viral hepatitis even if the level of evidence is low.^107^^,^^108^

Haemangioendothelioma was described in 1982 as a vascular neoplasia affecting different organs, with a prevalence of less than 1 per million.[109], [110], [111] The most common organs involved are the liver alone (21%), the liver and lung (18%), the lung alone (12%) and bone alone (14%), but any site in the body can be affected.^107^^,^^112^ HEH is more frequent in females (61% to 80%).^107^^,^^110^ The clinical behaviour is heterogeneous, ranging from indolent to aggressive behaviour.^112^

#### Pathophysiology

The *CAMTA1-WWTR1* gene fusion, resulting from a translocation t(1;3)(p36.3;q25) involving *CAMTA1* (a calmodulin-binding transcription activator) and *WWTR1* (coding for TAZ – a transcriptional coactivator) is pathognomonic of HEH (Table 2).^113^ *In cellulo*, *CAMTA1-WWTR1* fusion results in nuclear localisation of the fusion protein and leads to constitutive activation of the hippo pathway through a TAZ-dependent transcriptomic programme.^114^ Around 90% of HEHs harbour the *CAMTA1-WWTR1* gene fusion which has been consistently identified in haemangioendothelioma, irrespective of the primary site.^115^ Moreover, a rare *YAP1-TFE3* fusion has been identified in HEHs without *CAMTA1-WWTR1* fusion (Table 2).^116^^,^^117^ Detection of *CAMTA1-WWTR1* fusion by FISH or RT-PCR, or nuclear CAMTA1 expression at immunohistochemistry, is useful to confirm the diagnosis of HEH, as this fusion has not been identified in other human tumours.^117^^,^^118^

#### Diagnosis

A systemic review including 402 patients with HEH reported that 25% were asymptomatic, while right upper quadrant pain (48.6%), hepatomegaly (20.4%) and weight loss (15.6%) were the most frequent symptoms at diagnosis. Extrahepatic metastases were observed in 36.6% of patients.^108^ HEH could be nodular or diffuse and nodular lesions are usually multiple and affect both lobes of the liver.

A HEH should be suspected in cases of multifocal nodules (88%), which are sometimes coalescent, or the presence of nodules in subcapsular regions (up to 96%) with a capsular retraction (50 to 80%) on imaging.[119], [120], [121] Presence of ring enhancement at the tumour periphery on arterial phase is observed in 33% of patients, with a target appearance on the portal venous phase in 69% of cases – explained by central fibrosis with a concentric layer of tumour cells and a peripheral avascular rim on histology. On MRI, HEH harboured a target appearance on the T2-weighted sequences in 67% and on the diffusion-weighted sequences in 61% of patients.^121^

Histology is the gold standard for the diagnosis of HEH, with the help of immunohistochemistry (endothelial markers: factor VIII-related antigen, CD34 and CD31). The differential diagnosis with hepatic angiosarcoma is sometimes difficult to perform at histology and identification of *CAMTA1-WWTR1* fusion could be useful to confirm the diagnosis of HEH.^122^

#### Treatment

Therapeutic options in HEH depend on tumour burden, extrahepatic metastasis, resectability, age and comorbidities. The pattern of progression (stability *vs.* slow or rapid progression) should also be used to guide therapeutic decisions.

A comprehensive review of the literature reported the use of LT in 44.8% of patients, followed by no treatment in 24.8%, chemotherapy or radiotherapy in 21% and liver resection in 9.4%.^108^ Results from a multicentre database showed that LT led to a 5-year overall survival rate of 77.2% in 131 patients with HEH.^123^ Moreover, patients with extrahepatic metastasis could achieve prolonged survival after LT (up to 78% at 10 years).^124^

Risk factors for recurrence after LT were macrovascular invasion, waiting time of less than 3 months and lymph node metastases.^125^ A retrospective study suggested that similar outcomes could be achieved with resection or LT in HEH, although more patients at advanced stages were treated by LT^126^ As HEH is often a bilobar disease rarely amenable to liver resection, LT might be the best option even for patients with extrahepatic metastasis.^127^

In non-resectable or non-transplantable patients, different systemic treatments such as interferon alpha, thalidomide, doxorubicin, intra-arterial 5-fluorouracil and bevacizumab have been used in a very limited number of cases.^122^ In a pilot study of 15 patients affected by HEH of different localisation, sorafenib was associated with a median progression-free survival of 6 months.^128^ In a subset of patients with indolent disease, careful follow-up can be an option, with recent data reporting 10-year overall survival of 41% in selected patients.^129^^,^^130^ As no systemic treatment is currently approved for the treatment of HEH, a better understanding of the biological consequences of *CAMT1-WWTR1* fusion is needed to identify new therapeutic targets.

### Hepatic angiosarcoma

**Fig. 5:**
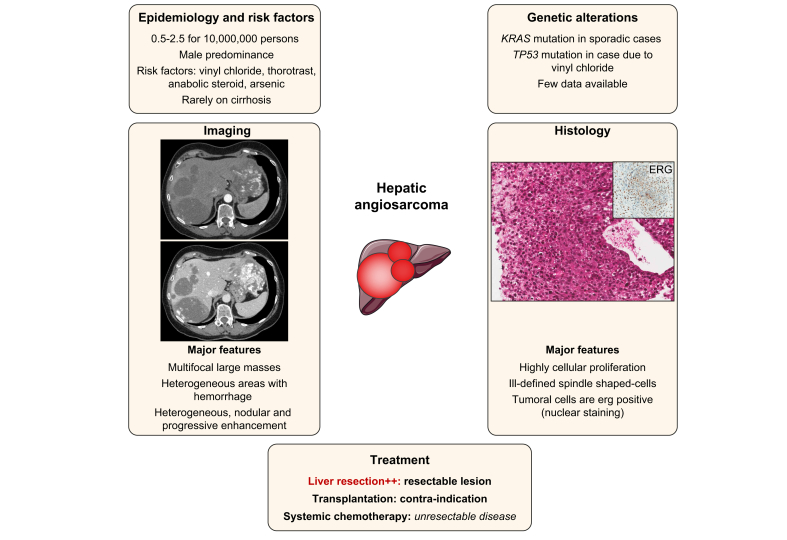
Main characteristics of hepatic angiosarcoma. Representation of the main genetic alterations, as well as clinical, histopathological, and radiological features of hepatic angiosarcoma; treatment strategies are also shown.

HAS is a high-grade aggressive mesenchymal malignancy, defining a subtype of soft-tissue sarcoma, composed of malignant endothelial cells of vascular or lymphatic origin that develop mostly on normal liver (Fig. 5). HAS is extremely rare, with an incidence estimated at 0.5–2.5 cases per 10,000,000 people, and more commonly develops in males (ratio 3:1).[131], [132], [133] In the 60s, 25% of HASs were associated with environmental risk factors such as vinyl chloride monomer, thorotrast, anabolic steroids and arsenic.^134^ When associated with vinyl chloride monomer, HAS can develop on cirrhosis (up to 20% to 43%).[135], [136], [137] Notably, the incidence of HAS declined following controls on vinyl chloride exposure in workers in the 70s.^138^^,^^139^

#### Pathophysiology

Overall, few data on molecular analysis are currently available: *KRAS* mutations have been described in sporadic cases, *TP53* mutations in vinyl chloride-related HAS, and recently a *ROS1-GOPC/FIG* fusion has been identified in 1 HAS.[140], [141], [142]

#### Diagnosis

Most of the time, patients with HAS have symptoms at presentation such as abdominal pain, fatigue, weight loss, hepatosplenomegaly, ascites, jaundice, and anaemia. The intraperitoneal rupture of HASs has been reported in 15–27% of patients.^143^ HASs have a very aggressive behaviour; poor prognostic factors are older age, large tumour size and high Ki-67 index.^131^^,^^133^ In a recent systematic review of 219 patients, the average age at onset was 56.7 years and distant metastases were frequent. The median overall survival was 6 months, with a 2-year survival rate of 17.3%.^144^

At contrast-enhanced imaging, HAS is usually multifocal with heterogeneous patterns, such as a progressive enhancement without washout at the portal and delayed phase. Progressive centripetal or diffuse “flash-fill” enhancement pattern (“reverse haemangioma”) with centrifugal enhancement have also been reported.^136^^,^^145^ HASs often contain haemorrhagic areas resulting in heterogeneous lesions on MRI, with hyperintense zones on T1WI and hypointense zones on T2WI.^145^

Some controversy exists about the performance of liver biopsy and about a potential high risk of bleeding .^137^^,^^143^^,^^146^ However, histology remains the gold standard for the diagnosis of HAS and liver biopsy is required to confirm the diagnosis.^133^ HAS is heterogeneous at histology, ranging from well-defined anastomotic vessels (vasoformative) to solid sheets of epithelioid or spindled cells without vasoformation, with different patterns sometimes mixed in the same tumours.^131^ HASs express ERG (erythroblast transformation specific-related gene) and endothelial markers such as CD31 and CD34.^147^

#### Treatment

Surgical resection seems the best therapeutic option, leading to a median overall survival of between 17 and 19 months.^143^^,^^148^ Survival was limited in studies assessing LT (around 6 months), with most of patients dying from tumour recurrence, explaining why HAS is a contraindication to LT.^149^^,^^150^ It is important to note that only 30% of patients had a known pre-LT diagnosis of HAS.^126^

Transarterial embolisation is frequently used to treat tumour bleeding with a limited impact on survival.^144^ There is no approved chemotherapy regimen for non-resectable liver HAS. ESMO guidelines on sarcomas report that angiosarcomas in general are sensitive to taxanes, reporting gemcitabine as an option alone or in combination with docetaxel.^151^

In a phase II trial including 3 primary liver angiosarcomas in patients with metastatic or unresectable disease, weekly paclitaxel led to progression-free survival rates of 74% and 45% at 2 and 4 months, respectively, with a median overall survival of 8 months.^152^ Palliative chemotherapy, such as 5-FU with doxorubicin or ifosfamide, carboplatin, bevacizumab or sorafenib, has been used in case reports or small series with limited radiological response and poor survival.^133^ Due to the rarity of this cancer, the management of HAS should be made in centres with multidisciplinary expertise on sarcomas.

## Conclusion

Despite several advances in recent decades, mainly in the field of pathophysiology, the diagnosis of rare PLCs remains challenging, and the prospective collection of dedicated clinical data, as well as trial recruitment, remain limited. Moreover, grants dedicated to these PLCs are lacking and pharmaceutical companies are rarely interested in the development of new drugs for these patients. To bypass these limitations, large international consortiums are needed to raise grants to run large prospective cohorts and to better define rare PLCs in terms of pathophysiology and clinical behaviour. This cooperative network will also be the basis of future clinical trials.

## Financial support

The authors received no financial support to produce this manuscript.

## Authors' contributions

Elia Gigante: writing, revision and approval of the manuscript. Valérie Paradis: writing, revision and approval of the manuscript. Maxime Ronot: writing, revision and approval of the manuscript. François Cauchy: writing, revision and approval of the manuscript. Olivier Soubrane: revision and approval of the manuscript. Nathalie Ganne-Carrié : writing, revision and approval of the manuscript. Jean-Charles Nault: writing, revision and approval of the manuscript.

## Conflicts of interests

Jean-Charles Nault received research grant from 10.13039/100004326Bayer for 10.13039/501100001677Inserm UMR1148, Nathalie Ganne-Carrié received personal fees from Bayer, Gilead, Ipsen and Shionogi. Elia Gigante, François Cauchy, Maxime Ronot, Valérie Paradis, Olivier soubrane: none to declare.

Please refer to the accompanying ICMJE disclosure forms for further details.

## Footnotes

Supplementary data to this article can be found online at https://doi.org/10.1016/j.jhepr.2020.100174.
