# Supplementary material for: New insights into the pathophysiology and clinical care of rare primary liver cancers
Source: JHEP Rep. 2020 Aug 24;3(1):100174. doi: 10.1016/j.jhepr.2020.100174 (PMC7653076; doi:10.1016/j.jhepr.2020.100174)
Supplement: Supplementary information.pdf [file mmc1.pdf]

New insights into the pathophysiology and clinical care of rare primary liver cancers

Elia Gigante, Valérie Paradis, Maxime Ronot, François Cauchy, Olivier Soubrane, Nathalie Ganne-Carrié, Jean-Charles Nault

Table of contents

Table S1……………………………………………………………………………………2

Table S1: different classifications of hepatocholangiocarcinoma

**Allen et Lisa 1949** (1)	<u>(A)</u> Double tumor with distance between the two lesions.<br><u>(B)</u> Combined type in which both HCC and iCCA components show contiguity with merging.<br><u>(C)</u> Mixed type in which the two neoplasias show a strict contact with a probable origin from the same site.
**Goodman 1985** (11)	<u>Type I</u> or "collision tumor," separate lesions of HCC and iCCA arising in the liver of the same patient.<br><u>Type II</u> or "transitional tumors" with elements of both HCC and iCCA in the same tumor with areas of transition.<br><u>Type III</u> or "fibrolamellar tumors" similar to the fibrolamellar variant of HCC with mucin-producing pseudo glands.
**WHO 2010** (157)	<u>Classical type,</u> composed of HCC and iCCA with presence of transition zones.<br><u>Stem cell type</u> divided further in :<br>-Typical :<br>Presence of foci of mature hepatocytes enclosed by peripheral clusters of small cells with morphological and immunohistochemical characteristics of progenitor cells<br>-Intermediate Cells:<br>Presence of intermediary features between hepatocytes and cholangiocytes with immunohistochemical markers of both histological entities. Organization in trabeculae, nests or strands.<br>-CLC:<br>Presence of cells mimicking cholangioles organized in a tubular anastomosing (antler-like) pattern surrounded by a sclerotic stroma and expressing progenitor cell markers
**Consensus terminology 2018** (4)	<u>Combined HCC-CCA (cHCC-CCA)</u>: presence of hepatocytic and cholangiocytic histology mixed with a transition or separates areas within the same tumor.<br><u>Intermediate cell carcinoma:</u> small cells of size between stem cells and hepatocytes and morphology between hepatocytes and cholangiocytes, if this entity should be considered as a subtype of cHCC-CCA is still a matter of discussion.<br><u>Pure cholangiolocarcinoma (CLC)</u>, reclassified in CCA
